# ErisNet: A Deep Learning Model for Noise Reduction in CT Images

**DOI:** 10.3390/bioengineering12090997

**Published:** 2025-09-19

**Authors:** Fabio Mattiussi, Francesco Magoga, Andrea Cozzi, Salvatore Ferraro, Gabrio Cadei, Chiara Martini, Svenja Leu, Ebticem Ben Khalifa, Alcide Alessandro Azzena, Marco Pileggi, Ermidio Rezzonico, Stefania Rizzo

**Affiliations:** 1 Imaging Institute of Southern Switzerland (IIMSI), Ente Ospedaliero Cantonale (EOC), 6900 Lugano, Switzerland; francesco.magoga@eoc.ch (F.M.); salvatore.ferraro@eoc.ch (S.F.); gabrio.cadei@eoc.ch (G.C.); svenja.leu@eoc.ch (S.L.); ebticem.benkhalifa@eoc.ch (E.B.K.); alcidealessandro.azzena@eoc.ch (A.A.A.); ermidio.rezzonico@eoc.ch (E.R.); stefania.rizzo@eoc.ch (S.R.); 2 Department of Medicine and Surgery, University of Parma, Maggiore Hospital, 43126 Parma, Italy; martinic@ao.pr.it; 3 Diagnostic Department, Azienda Ospedaliero-Universitaria di Parma, 43126 Parma, Italy; 4 Strategic Steering Committee, Centro Studi SAPIS Foundation, Italian National Federation of Orders of Radiographers and Technical, Rehabilitation, and Prevention Health Professions Research Centre, Via Magna Grecia, 30/A, 00183 Roma, Italy; 5 Neurocentro della Svizzera Italiana (NSI), Ente Ospedaliero Cantonale (EOC), 6900 Lugano, Switzerland; marco.pileggi@eoc.ch; 6 Faculty of Biomedical Sciences, Università della Svizzera italiana, 6962 Lugano, Switzerland

**Keywords:** neural network, CT images, noise reduction, deep learning, medical imaging

## Abstract

**Background**: ErisNet, a novel AI model to reduce noise in Computed Tomography images. **Methods**: We trained ErisNet on 23 post-mortem whole-body CT scans. We assessed the objective performance with mean square error (MSE), peak signal-to-noise ratio (PSNR), structural similarity index (SSIM) measure, visual information fidelity (VIF), edge preservation index (EPI) and noise variance (NV). We assessed the qualitative performance by six radiologists. To support the visual assessment, we placed circular regions of interest (ROI) in the vitreous body, brain, liver and spleen parenchyma and paravertebral muscle. **Results**: ErisNet achieved MSE 64.07 ± 46.81, PSNR 31.32 ± 3.69 dB, SSIM 0.93 ± 0.06, VIF 0.49 ± 0.09, EPI 0.97 ± 0.01 and NV 64.69 ± 46.80. The ROI analysis showed a reduction in noise: the SD of the HU decreased by 8% in the vitreous body (from 17.6 to 16.2 HU), by 18% in the brain parenchyma (from 18.85 to 15.40 HU) and by 15–19% in the liver, spleen and paravertebral muscle. The six radiologists confirmed these results by assigning high scores (scale from one to five): overall quality 4.5 ± 0.6, noise suppression/detail preservation 4.7 ± 0.5 and diagnostic confidence 4.8 ± 0.4 (*p* < 0.01). **Conclusions:** ErisNet improves the quality of CT images and shows strong potential for processing low-dose scans.

## 1. Introduction

Images obtained using Computed Tomography (CT) play a crucial role in medical diagnosis, allowing for a detailed visualization of the internal structures of the human body [1]. However, the presence of noise can compromise the quality of these images, which can limit the diagnostic ability by increasing the risk of erroneous interpretations [2].

This issue is closely related to the amount of voltage and current, kV and mA, respectively, utilized during volume scanning; a lower use of these parameters results in a lower radiological dose delivered to the patient. The continuous development of noise reduction techniques [3] is essential to maintain or improve the diagnostic quality of CT images [4].

Previous studies have taken numerous approaches to noise reduction, ranging from traditional filtering techniques or iterative reconstructions [5] to more recent machine learning models [6]. Sadia et al. [7] provided a comprehensive review of CT image denoising methods, highlighting that deep learning-based approaches have shown significant promise for enhancing image quality while enabling a reduction in radiation dose. For example, Chen et al. [8] developed an iterative denoising technique that exploits the spatial redundancy of data in the direction of increasing slice thickness, tested on phantom experiments. Liu et al. [9] proposed a framework that integrates a physical model with an unsupervised learning approach, using CT scans of phantom at different doses to train generative neural networks. In addition, Won et al. [10] presented a self-supervised learning method for denoising CT images by generating pseudo-TC image pairs by adding artificial noise. Zhang et al. [11] introduced an unsupervised method that exploits the similarity between adjacent slices in thin-layer CT scans, using data with artificial noise to train the network. Finally, Zainulina et al. [12] proposed a self-supervised physics-based denoising approach, which does not require high-dose CT images as the ground truth, employing simulated noise for training. Although these approaches have shown promising results, many of them are based on synthetic or phantom data, which may not fully represent the complexities and variabilities present in real clinical scans. Furthermore, recent benchmarking studies by Eulig et al. [13] revealed that most deep learning-based denoising methods show a statistically similar performance, with marginal improvements over the past years, highlighting the need for more rigorous evaluation approaches and novel methodologies that can achieve meaningful advances in the field.

In this study, we chose to use post-mortem CT (PMCT) scans to develop and validate our model. This choice allowed us to acquire CT images paired with both high and low radiation doses from the same subject, thus generating corresponding image pairs with different noise levels. Such an approach would not be feasible in living patients due to ethical and radiation protection constraints. Furthermore, PMCT provides images of the entire body (from head to toe), offering a diverse anatomical dataset for training and evaluation.

The techniques and limitations of these previous studies are summarized in Table 1, highlighting the need for approaches that can be validated on real clinical data while maintaining the balance between noise reduction and diagnostic quality preservation.

The main goal of ErisNet is to significantly reduce noise in CT images while preserving essential diagnostic quality, to facilitate diagnoses with greater accuracy and potentially reduce the radiological dose administered to patients.

While methods such as U-Net [14] and denoising convolutional neural networks (DnCNN) [15] have shown promising results, they often require a trade-off between noise suppression and preservation of diagnostic details.

ErisNet builds on these assumptions by integrating an encoder–decoder architecture, inspired by U-Net, with advanced denoising strategies, thereby improving performance and overcoming limitations of existing models by offering superior noise reduction without compromising image clarity.

The purpose of this study was to develop and validate ErisNet, demonstrating its potential in reducing noise in CT images. By effectively reducing noise, ErisNet aims to improve image quality and support patient safety by enabling the use of lower radiation doses without compromising diagnostic accuracy.

The manuscript is organized as follows:

1. Materials and Methods; describes the postmortem CT dataset comprising 23 whole-body scans, the detailed architecture of ErisNet integrating encoder–decoder blocks with DnCNN components, hyperparameter optimization procedures, and evaluation metrics including quantitative image quality measures and qualitative radiological assessment protocols.
2. Results: presents the complete experimental results related to three evaluation approaches: global quantitative analysis using six image quality metrics (MSE, PSNR, SSIM, VIF, EPI, NV), organ-level ROI analysis in five anatomical locations, and qualitative evaluation by six expert radiologists using a structured nine-question assessment.
3. Discussion: Provides an in-depth analysis of the results, a comparison with existing denoising methods, the architectural advantages of the proposed approach, and contextualizes the results within the current literature.
4. Conclusion: Summarizes the limitations of the study, outlines future research directions, and presents key conclusions regarding the potential of ErisNet for reducing clinical CT dose while maintaining diagnostic quality.

## 2. Materials and Methods

The local Ethics Committee (Comitato Etico Cantonale—Repubblica e Cantone Ticino, Bellinzona, Switzerland) determined that ethical approval was not required according to Swiss law (Req-2024-00458).

### 2.1. Dataset

For the training and evaluation of the ErisNet model, a dataset comprising 23 whole-body CT scans (head-to-toe) obtained from PMCT examinations was used. According to local protocols for PMCT, these scans were anonymously acquired between November 2023 and March 2025 at the Imaging Institute of Southern Switzerland (IIMSI), Ente Ospedaliero Cantonale (EOC), Lugano, Switzerland, using two 128-slice CT scanners (Somatom Definition Edge and Somatom X.cite, Siemens Healthineers; Erlangen, Germany).

**Figure 1:**
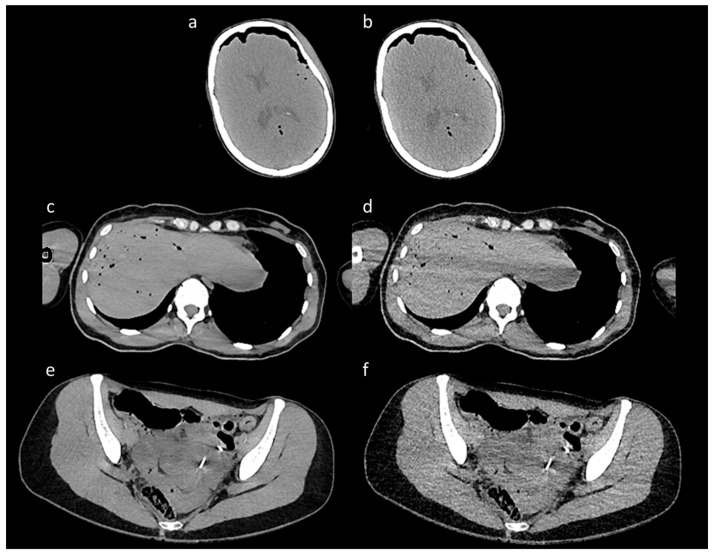
Representative paired CT images from the training set. The figure shows a representative selection of paired CT images from the training dataset. The figure is organized into three pairs, each featuring a high-dose image and its corresponding low-dose counterpart. The high-quality images, shown in panels (**a**,**c**,**e**), served as clean reference targets for the model, while the LD images in panels (**b**,**d**,**f**) were used as noisy inputs. The paired images come from three distinct anatomical regions: (**a**,**b**) show a scan of the skull, (**c**,**d**) show a scan of the abdomen at the level of the liver, and (**e**,**f**) illustrate a scan of the pelvis.

The scans encompassed a range of body dimensions representative of diverse patient anatomies, with heights ranging from 142 cm to 204 cm and varying body compositions, including a broad spectrum of body mass indices (BMI 18–32). The CT images were reconstructed using the Iterative Reconstruction technique, generating pairs of high-quality (HQ) and low-quality (LQ) images for each scan (Figure 1). Specifically, low-dose (LQ) scans exhibited significant noise levels, while high-dose (high-quality) scans served as a reference standard with low noise.

Each CT scan produced approximately 3300 images, with slice thicknesses of 0.6 mm and a spatial resolution of 512 × 512, equally divided between HQ and LQ. The mean value of dose reduction in the LQ scans was 55% (minimum 41%–maximum 83%), allowing a significant decrease in X-ray exposure without compromising the detail and contrast scale of the images for diagnostic analyses.

Table 2 depicts the dose indices Volume Computed Tomography Dose Index (CTDIvol) and Dose Length Product (DLP) relating to each of the 23 scans (from 1 to 6: PMCT examinations using the Definition Edge model; From 7 to 23: PMCT examinations using the X.cite model), including specifics on the variation in exposure parameters used to create variability in the LQ scans.

#### Dataset Management and Data Augmentation

The dataset was divided into three distinct subsets: the training, validation, and test sets. Specifically, 15 patients were allocated to the training set, four to the validation set, and the remaining four patient were used for the test set. The data augmentation process, consisting of random flips and rotations, was applied exclusively to the training set to enhance the diversity and volume of data available for network training.

In our data augmentation process, we used standard transformations provided by PyTorch’s torchvision. transforms library. The operations are random rotation (with a range of ±10 degrees) and random flipping (both horizontal and vertical, with a probability of *p* = 0.5).

This strategy aims to improve the model’s robustness and efficacy [16] by exposing it to a diverse range of data variations during the training phase. The test and validation sets were excluded from the data augmentation process. This decision was made to ensure that the performance evaluation of the model was conducted on unmodified data, more accurately reflecting real-world conditions and maintaining the integrity of the evaluation approach.

By implementing a data augmentation factor of four, we substantially increased the number of images available for training. Specifically, the training set was expanded to contain ~198.000 LQ images and an equal number of HQ images. This increase in data volume also mitigates the risk of overfitting.

### 2.2. ErisNet Model Architecture

**Figure 2:**
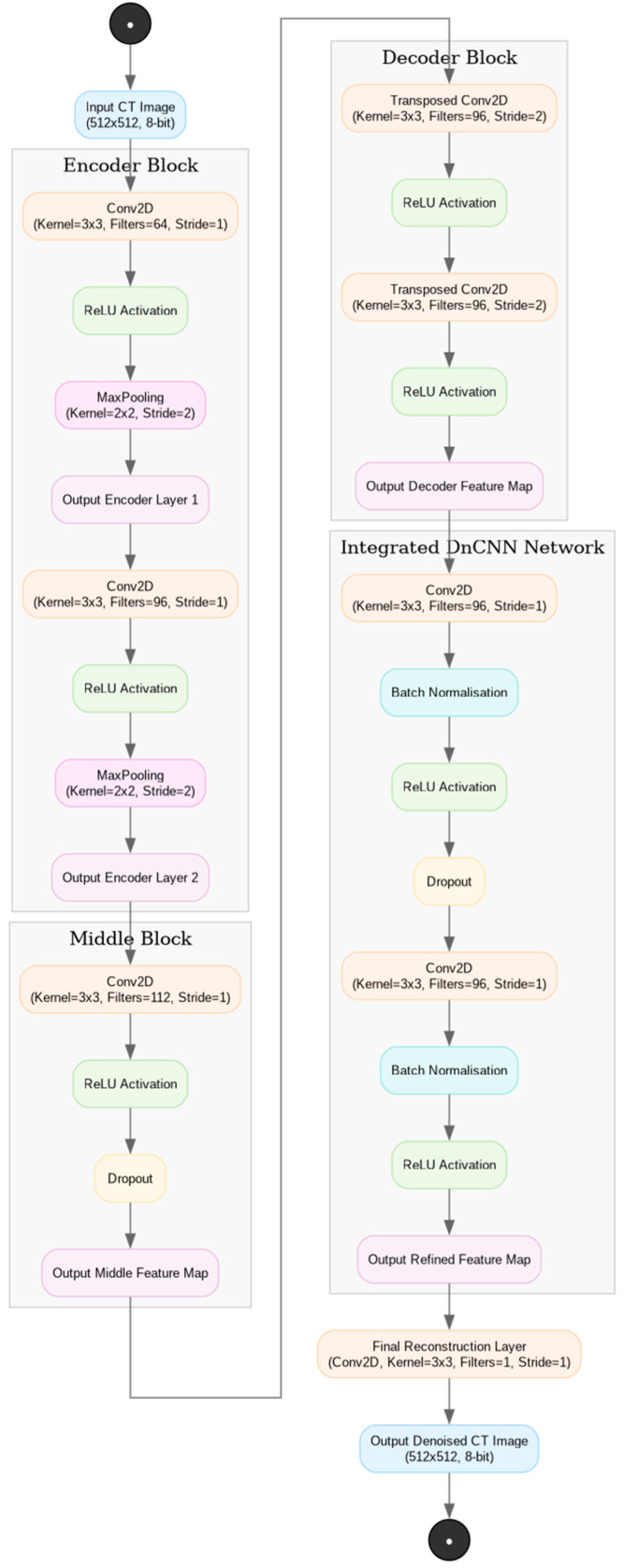
ErisNet architecture for CT image denoising. The diagram shows the data flow from the CT input (512 × 512, 8-bit) through four main blocks. The Encoder block contains two stages of convolution (Conv2D), ReLU activation, and pooling that progressively reduce the resolution with 64 and 96 filters. The Middle block applies convolution with 112 filters, ReLU and dropout as the bottleneck of the architecture. The Decoder block reconstructs the resolution using transposed convolutions with 96 filters and ReLU activations. Finally, the integrated DnCNN block refines the features through two convolutions with batch normalization, ReLU and dropout. The architecture ends with a final reconstruction layer (Conv2D with 1 filter) that produces the denoised CT image at the output, effectively combining spatial encoding/decoding with specialized refinement for noise reduction.

The ErisNet model (Figure 2) integrates the capabilities of the encoder–decoder architecture, inspired by U-Net [17], with the efficacy of a denoising convolutional neural network (DnCNN) for noise removal [18]. This integration aims to mitigate noise in CT images while preserving critical diagnostic details.

The ErisNet encoder is designed to extract essential features of CT images while reducing their spatial dimensions. It employs 2D convolutions with Rectified Linear Unit (ReLU) activation and pooling to process the images, retaining pertinent information while diminishing the spatial dimensions. This phase progressively increases the number of feature channels.

The middle block operates on an intermediate level of feature representation, implementing a 2D convolution to further augment the number of channels. The incorporation of a dropout layer at this stage aims to mitigate overfitting, enhancing the model’s robustness and generalizability.

In the decoder, the model restores the image dimensions to their original values utilizing 2D transposed convolutions. This process reduces the number of channels while increasing the spatial dimensions of the image. The reintroduction of features captured by the encoder through skip connections [19] facilitates the preservation of salient details in images.

Integrated into ErisNet, the DnCNN network specializes in noise reduction. Through a series of 2D convolutions, batch normalization, ReLU activations, and dropout, this network refines the decoder’s output, effectively attenuating noise from CT images.

The input to the neural network is in DICOM format (Digital Imaging and COmmunications in Medicine), with a spatial resolution of 512 × 512 and a pixel depth of 8 bits.

### 2.3. ErisNet Model Mathematical Details

Mathematical formulas were adopted for each key step in the ErisNet model to justify its operation:

- 2D convolution operation: $y\left(i,j\right)=\sum_{m,n}x\left(i+m,j+n\right)\cdot w\left(m,n\right)+b$ where x represents the input image, w the kernel (filter) applied, b the bias, and $y\left(i,j\right)$ the resulting value at position $\left(i,j\right)$;
- ReLU Activation Function: $f\left(x\right)=max(0,x)$ which keeps the positive values and puts the negative ones to 0;
- Max pooling operation: y(i,j)=max(p,q)∈kenrelx(i+p,j+q) where x(i+p,j+q) represents the value of the pixel in the feature map at the position (i+p,j+q) within the kernel window, i,j indicate the coordinates (row, column) of the current position in the output image after pooling is applied, (p,q): are the offsets within the kernel window, the kernel represents the local area over which max pooling is calculated,
- Batch normalization: $\hat{x}=\frac{x-\mu }{\sqrt{\sigma^{2}+\epsilon }}$ where μ and σ are the mean and variance of the batch, and ϵ is a small stabilization term;
- Dropout used to reduce overfitting, randomly cancels a fraction of the nodes during training, reducing feature co-adaptation. Mathematically, we multiply each activation by a binary variable d (with probability *p* to be 0) during training.

### 2.4. Hardware and Software Configuration

The network training was conducted on a system equipped with Ubuntu 24.04 Operating System, an Intel Core i9 14900 k processor, 64 GB of DDR5 RAM and an NVIDIA RTX 4090 GPUs with a total of 24 GB of VRAM. The model was implemented utilizing Python 3.12.3, PyTorch 2.3.0, NVIDIA drivers 570.124.06, NVIDIA CUDA Toolkit 12.8.

### 2.5. Hyperparameter Optimization

To identify the optimal hyperparameter configuration for our neural network, we employed Hyperopt, a Python library for hyperparameter optimization, through Bayesian search algorithms. The search space encompassed the dropout rate (from 0.2 to 0.6), the number of channels in the encoder layers (64, 96, or 128), middle layer (64, 96, or 128), decoder (64, 96, or 128), the number of DnCNN blocks (2, 4, 6, or 8), the learning rate (log-uniform distribution between 10^−6^ and 10^−5^), and the batch size (2, 4, 6, or 8).

We defined an objective function to minimize training loss using Mean Squared Error as the cost function. Hyperopt’s Tree-structured Parzen Estimator algorithm was used to perform 100 hyperparameter evaluations, with the optimal configuration saved in a JSON file. Following identification of the hyperparameters that yielded the best performance, we conducted a fine-tuning phase to further refine these values. This more targeted search was executed to enhance the model’s stability and robustness by mitigating the risk of overfitting. We narrowed the search space around the optimal hyperparameters by examining small variations to ascertain their impact on performance.

The fine-tuning search space included the dropout rate (from 0.4 to 0.5), the number of channels in the encoder layers (64, 96, or 112), middle layer (96, 112, or 128), decoder (64, 96, or 112), the number of DnCNN blocks (3, 4, or 5), the learning rate (log-uniform distribution between 10^−5^ and 10^−4^), and the batch size (4, 6, or 8). This approach enabled us to obtain a configuration that not only minimizes loss but is also resilient to minor variations, ensuring the model is robust and reliable in real-world scenarios. The final optimal hyperparameter configuration obtained from fine-tuning was batch size (6), learning rate (0.003), dropout rate (0.5), number of channels in the encoder layers (96), number of channels in the middle layer (112), number of channels in the decoder layers (96), number of DnCNN blocks (4).

### 2.6. Evaluation Metrics

In this study, the following metrics were employed to evaluate the model’s performance in noise reduction:

*Mean squared error (MSE):* utilized to quantify the average squared difference between the HQ images and the corresponding images processed by the model.

$$MSE=\frac{1}{mn}\sum_{i=1}^{m}\sum_{j=1}^{n}{\left(I_{HQ}\left(i,j\right)-I_{output}\left(i,j\right)\right)}^{2}$$

- *m:* number of rows (height) of the image.
- *n:* number of columns (width) of the image.
- $I_{HQ}\left(i,j\right)$*:* pixel value in the HQ image at position (*i*,*j*).
- $I_{output}\left(i,j\right)$*:* pixel value in the processed image at position (*i*,*j*).

*Peak signal-to-noise ratio (PSNR)* [20]: provides a measure of the signal-to-noise ratio. It was calculated to evaluate the efficacy of the model in improving the visual quality of the CT images.

$$PSNR=10{log}_{10}\left(\frac{L^{2}}{MSE}\right)$$

- *L:* maximum possible pixel value, which is 255 (8-bit images).

*Structural similarity index measure (SSIM)* [21]: evaluates the structural similarity between HQ images and processed images, considering luminance, contrast, and structure.

$$SSIM\left(x,y\right)=\frac{\left(2\mu_{x}\mu_{y}+C_{1}\right)\left(2\sigma_{xy}+C_{2}\right)}{\left(\mu_{x}^{2}+\mu_{y}^{2}+C_{1}\right)\left(\sigma_{x}^{2}+\sigma_{y}^{2}+C_{2}\right)}$$

- $\mu_{x}$*:* mean of the pixel values in the HQ image.
- $\mu_{y}$*:* mean of the pixel values in the processed image.
- $\sigma_{x}^{2}$*:* variance of pixel values in HQ image.
- $\sigma_{y}^{2}$*:* variance of the pixel values in the processed image.
- $\sigma_{xy}$*:* covariance of the pixel values in the two images.
- *C*_1_ and *C*_2_ are constants to avoid division by zero.

*Visual information fidelity (VIF)* [22]: utilized to quantify the fidelity of visual information retained in a reduced noise-image compared to the original image.

$$VIF=\frac{\sum_{s=1}^{S}I\left(W_{s};W_{s}\vee F_{s}\right)}{\sum_{s=1}^{S}I\left(W_{s};E_{s}\vee F_{s}\right)}$$

- *S:* total number of sub-bands.
- $I\left(W_{s};W_{s}\vee F_{s}\right)$*:* mutual information between the original signal *Ws* and the distorted signal *Ŵs* given the filter coefficients *F_s_*
- $I\left(W_{s};E_{s}\vee F_{s}\right)$*:* mutual information between the original signal *Ws* and noise *Es*, given the filter coefficients *F_s_.*

*Edge preservation index (EPI)* [23]: employed to evaluate the efficacy of ErisNet in maintaining the edges and structural details of the original processed image. It is calculated as the ratio of the summation of the squared differences in the gradients of the original and noise-reduced images to the summation of the squared gradients of the original image.

$$EPI=\frac{\sum_{i,j}\left[{\left(\frac{\partial I_{HQ}\left(i,j\right)}{\partial x}-\frac{\partial I_{output}\left(i,j\right)}{\partial x}\right)}^{2}+{\left(\frac{\partial I_{HQ}\left(i,j\right)}{\partial y}-\frac{\partial I_{output}\left(i,j\right)}{\partial y}\right)}^{2}\right]}{\sum_{i,j}\left[{\left(\frac{\partial I_{HQ}\left(i,j\right)}{\partial x}\right)}^{2}+{\left(\frac{\partial I_{HQ}\left(i,j\right)}{\partial y}\right)}^{2}\right]}$$

- $\frac{\partial I_{HQ}\left(i,j\right)}{\partial x}$ gradient of the HQ image with respect to the x-coordinate at position (*i*,*j*).
- $\frac{\partial I_{output}\left(i,j\right)}{\partial x}$ gradient of the processed image with respect to the x-coordinate at position (*i*,*j*).
- $\frac{\partial I_{HQ}\left(i,j\right)}{\partial y}$ gradient of the HQ image with respect to the y-coordinate at position (*i*,*j*).
- $\frac{\partial I_{output}\left(i,j\right)}{\partial y}$ gradient of the processed image with respect to the y-coordinate at position (*i*,*j*).

*Noise Variance (NV):* quantifies the variance of noise in the HQ images and those processed by ErisNet to assess the extent of noise present in the images.

$$NoiseVariance=\frac{1}{mn}\sum_{i=1}^{m}\sum_{j=1}^{n}{\left(I\left(i,j\right)-\mu \right)}^{2}$$

- *m:* number of rows (height) of the image.
- *n:* number of columns (width) of the image.
- $I\left(i,j\right)$*:* pixel value in the image at position $\left(i,j\right)$.
- *μ:* mean of pixel values in the image.

### 2.7. ROI Evaluation

**Figure 3:**
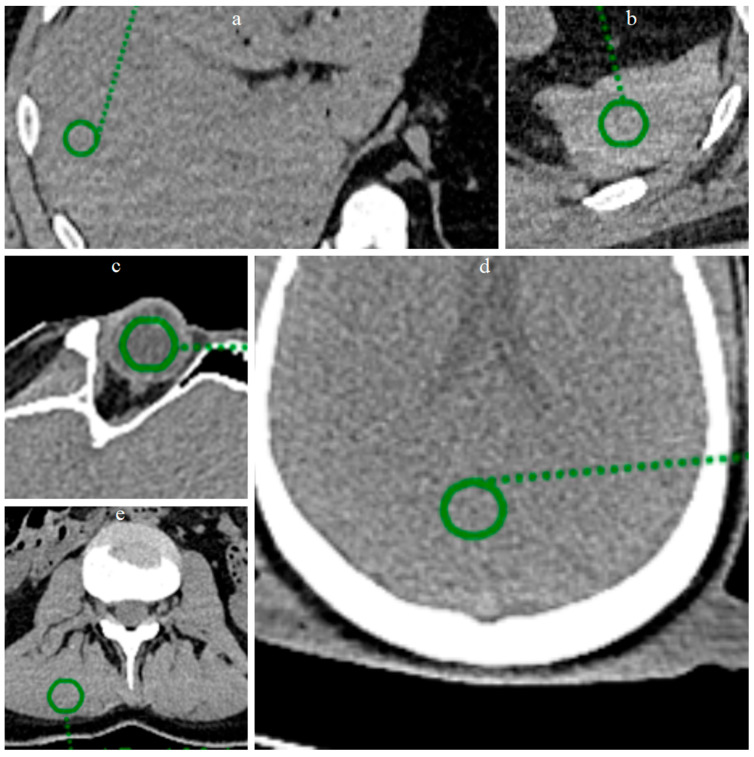
ROI placement at five anatomical locations for quantitative analysis. Circular green ROIs with radius 8 mm were positioned on: (**a**) liver parenchyma, (**b**) splenic parenchyma, (**c**) vitreous body of the eye, (**d**) brain parenchyma, and (**e**) lumbar paravertebral muscle. These anatomical regions were selected to evaluate ErisNet performance across different tissue types and attenuation characteristics.

In the present study, a quantitative noise analysis was also performed by applying circular regions of interest (ROI) (radius 8 mm) at five anatomical locations (Figure 3): vitreous body, brain parenchyma, liver parenchyma, splenic parenchyma and paravertebral muscle. Within each ROI, the mean and standard deviation (SD) of the attenuation values (Hounsfield units) were calculated in order to characterize and compare the noise level before and after processing with ErisNet.

### 2.8. Qualitative Evaluation

The qualitative assessment was performed by six experienced radiologists who examined each image processed with ErisNet by answering a questionnaire based on a 5-point Likert scale (1 = lowest score, 5 = highest score). Specifically, scores were given on the following nine criteria:

- Overall assessment of the visual result.
- Ability of ErisNet to attenuate noise without altering diagnostic details.
- Preservation of anatomical detail, contrast and tissue differentiation.
- Presence of artefacts, possible occurrence of artefacts that interfere with the diagnostic interpretation.
- Diagnostic confidence, degree of support provided by the image to the clinical decision-making process.
- Noise reduction-detail balance, trade-off between noise dampening and fidelity of anatomical structures.
- Image naturalness, perception of realism and consistency with the expected anatomy, avoiding an excessively smooth or artificial appearance.
- Clinical usefulness, potential applicability of the images in daily clinical practice.
- Improvement over the original image, degree of improvement observable by comparing the processed images with unprocessed low-dose images.

The scores of each radiologist were collected and subsequently analyzed to objectively assess the impact of ErisNet on the diagnostic quality of PMCTs.

## 3. Results

**Figure 4:**
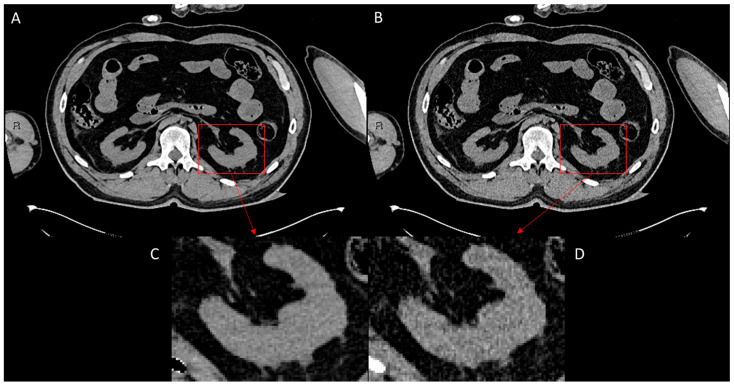
Comparison of CT image quality before and after processing with ErisNet, with detailed enlargements. (**A**) Image processed with ErisNet, showing significantly reduced noise levels and increased sharpness. (**B**) Corresponding low-dose image, used as input for ErisNet, showing increased noise. (**C**) Magnified view of a specific region of interest (red box), the left kidney, in image (**A**), highlighting the noise reduction and detail preservation achieved by ErisNet. (**D**) Magnified view of the same region of interest (red box) of the left kidney in image (**B**), illustrating the significant noise present in the original low-dose image before processing.

To evaluate the performance of ErisNet on the test set, we implemented a comprehensive protocol [24] to assess the model’s efficacy in noise reduction and its subsequent impact on CT image quality. The following results indicate ErisNet’s performance across various metrics, demonstrating a significant reduction in noise compared with the original LQ images (Figure 4).

### 3.1. Quantitative Results

ErisNet:

- Increased PSNR from an average of 30.78 in the LQ images to 31.32 in the ErisNet-processed images, with a SD of 3.69;
- Substantially reduced MSE from 73.45 in the LQ images to 64.70 in the ErisNet-processed images;
- Increased the SSIM values from 0.92 to 0.94;
- Increased VIF from 0.47 in LQ images to 0.49;
- Did not compromise EPI, which was 0.97 in LQ images and 0.97 with ErisNet;
- Decreased noise variance from 73.43 in LQ images to 64.69 in ErisNet;

#### 3.1.1. Violin Plots Description

**Figure 5:**
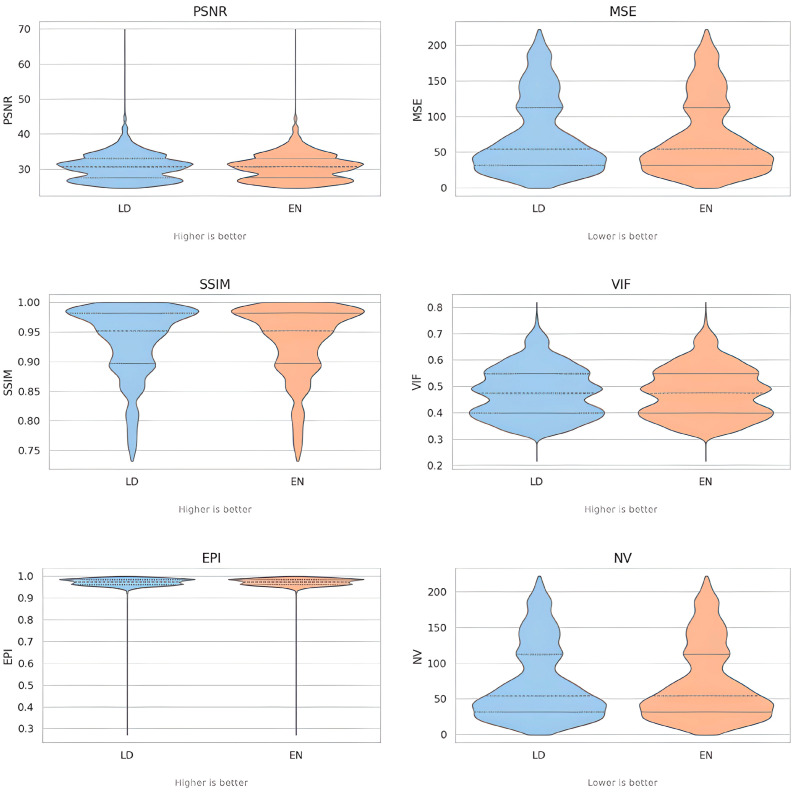
Violin plots showing the distribution of image quality metrics across the test dataset. The plots display the performance evaluation of ErisNet using six quantitative metrics: Peak Signal-to-Noise Ratio (PSNR), Mean Squared Error (MSE), Structural Similarity Index Measure (SSIM), Visual Information Fidelity (VIF), Edge Preservation Index (EPI), and Noise Variance (NV). The violin plots illustrate both the central tendency and variability of each metric.

Figure 5, which displays data distributions using violin plots, provides a visual analysis of the performance of the ErisNet model compared to the low-dose protocol. These plots show the density and consistency of the results for each metric. For noise metrics such as MSE and NV, ErisNet’s violin plot shows a distribution shifted towards lower values than LQ. The narrower and more compact shape of ErisNet’s ‘violin’ indicates that the model not only reduces noise on average, but does so more uniformly and predictably across all samples, with fewer extreme values. Similarly, for metrics that measure image quality and detail preservation such as PSNR, SSIM, VIF, and EPI, ErisNet’s violin plots are shifted towards higher values. Their distributions are more concentrated towards the higher end of the value range than those of LQ.

#### 3.1.2. Table 3 Description

Table 3 provides a quantitative summary of the key performance metrics used to evaluate our model. The table presents specific numerical values and distributions for each metric, including the mean, standard deviation, and a range of statistical values. This data provides a detailed analysis of the model’s performance under different conditions. The purpose of this table is to serve as a statistical reference for the results, allowing for direct analysis and comparison of the exact values of each performance metric.

**Table 3:** Global image quality metrics.

Metric	Mean	SD	Median	Min	Max
PSNR LQ	30.78	3.68	30.71	24.76	69.89
PSNR ErisNet	31.32	3.69	31.44	24.93	69.15
MSE LQ	73.45	52.91	55.12	0.06	217.05
MSE ErisNet	64.70	46.81	46.59	0.01	208.59
SSIM LQ	0.92	0.06	0.95	0.73	0.98
SSIM ErisNet	0.94	0.05	0.95	0.74	0.99
VIF LQ	0.47	0.09	0.47	0.21	0.78
VIF ErisNet	0.49	0.09	0.48	0.01	0.81
EPI LQ	0.97	0.01	0.97	0.27	0.99
EPI ErisNet	0.97	0.01	0.97	0.13	0.99
Noise Variance LQ	73.43	52.88	55.12	0.01	216.92
Noise Variance ErisNet	64.69	46.80	46.59	0.01	208.57

Table 3: Global image quality metrics (MSE, PSNR, SSIM, etc.) comparing low-quality scans, scans enhanced by ErisNet, and high-quality reference scans.

### 3.2. ROI Results

**Figure 6:**
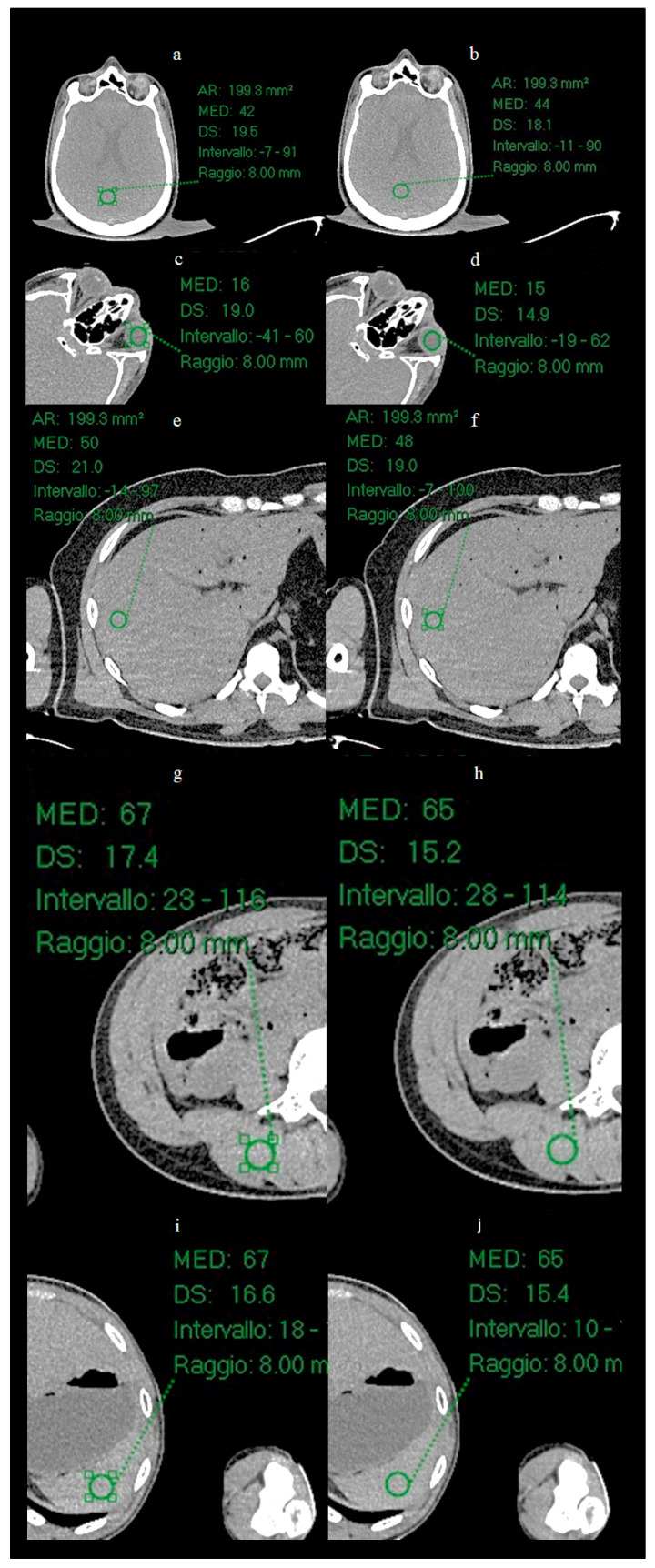
Positioning of ROIs in five anatomical locations for quantitative analysis. Circular ROIs with a radius of 8 mm were placed on: (**a**,**b**) brain parenchyma, (**c**,**d**) vitreous body of the eye, (**e**,**f**) liver parenchyma, (**g**,**h**) lumbar paravertebral muscle, and (**i**,**j**) spleen parenchyma. For each anatomical location, the image on the left shows the low-dose acquisition, while the image on the right shows the corresponding result processed by ErisNet. These anatomical regions were selected to evaluate ErisNet’s performance on different tissue types and attenuation characteristics. The comparison demonstrates a consistent reduction in the standard deviation of Hounsfield units within the ROIs after processing with ErisNet (Table 4).

Circular ROI of radius 8 mm were placed on the images of the Test Set on the vitreous body (Figure 6), brain parenchyma, liver parenchyma, splenic parenchyma and paravertebral muscle, and the mean and SD of the mean values of Hounsfield Units (HU) were calculated for each ROI.

The mean values of measured ROI are shown in Table.

**Table 4:** Organ level attenuation and noise.

Organ	Mean HU	SD	Difference HU (%)
Vitreous LQ	18.75	17.60	7.95
Vitreous ErisNet	18.00	16.20	
Brain LQ	45.50	18.85	18.30
Brain ErisNet	46.00	15.40	
Liver LQ	59.00	19.65	13.36
Liver ErisNet	58.00	17.03	
Spleen LQ	54.61	25.11	12.01
Spleen ErisNet	52.34	22.09	
Muscle LQ	52.12	25.65	9.35
Muscle ErisNet	50.74	23.26	

Table 4: Attenuation values (HU) and standard deviation at the organ level in five anatomical locations before (low dose) and after processing with ErisNet; the Difference (%) column shows the percentage reduction in noise.

### 3.3. Qualitative Results

Six radiologists examined the images produced by ErisNet. For Q1, the average is 4.5 (SD 0.55; raw *p* = 0.0011; Holm *p* = 0.0072) and the same value is repeated in Q2. The diagnostic sharpness assessed in Q3 rises to 4.67 (SD 0.52; raw *p* = 0.00052; Holm *p* = 0.00417), while the overall quality in Q9 reaches 4.83 (SD 0.41; raw *p* = 0.000108; Holm *p* = 0.000972). Noise reduction (Q4) scores 4.5 (SD 0.84; raw *p* = 0.00708; Holm *p* = 0.01415). The accuracy aspects measured in Q5 and Q6 stood at 4.33 (SD 0.52; raw *p* = 0.00146; Holm *p* = 0.00655). The lowest scores were for Q7, 3.83 (SD 0.41; raw *p* = 0.00410; Holm *p* = 0.01231), and Q8, also 3.83 (SD 0.75; raw *p* = 0.04219; Holm *p* = 0.04219).

All values are significantly above the neutral threshold (3/5). However, to assess the clinical relevance of these differences, we also considered the effect size using Cohen’s d. This analysis reveals that, despite some minor numerical differences, the perceived impact on radiologists varied significantly. For example, Q9 (Cohen’s d = 4.49), Q3 (Cohen’s d = 3.23) and Q1 and Q2 (Cohen’s d = 2.74) showed very high clinical relevance, indicating a substantial and tangible positive impact. Questions Q5 and Q6 (Cohen’s d = 2.58) and Q7 (Cohen’s d = 2.04) also demonstrated strong clinical relevance, while Q4 (Cohen’s d = 1.79) had a moderate effect. The lowest clinical relevance was found for question Q8 (Cohen’s d = 1.11), suggesting that its practical impact, although statistically significant, was less pronounced than the others. The complete statistics are shown in Table 5.

**Figure 7:**
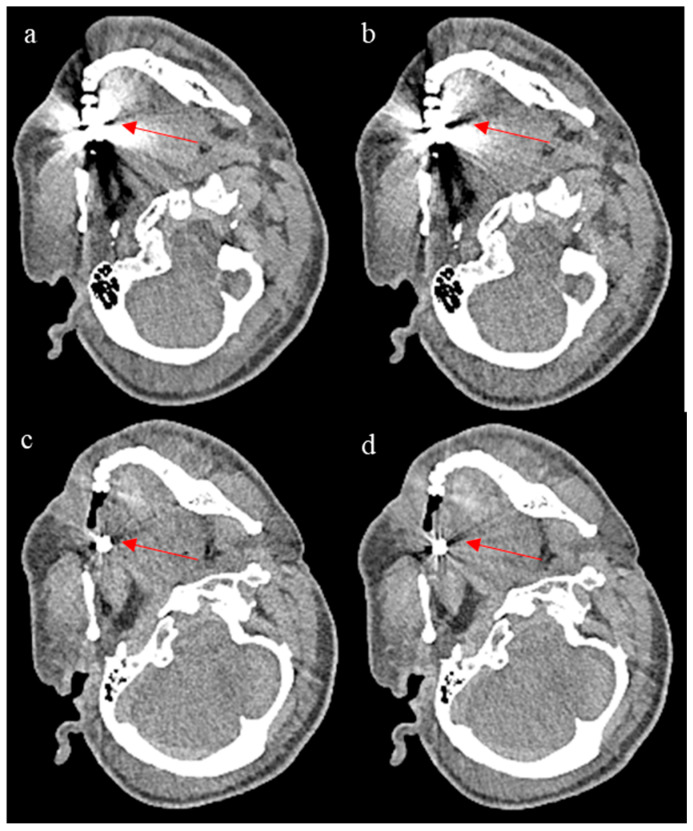
The figure shows ErisNet’s performance on CT images containing metallic artefacts. (**a**,**c**) are images processed with ErisNet showing noise reduction compared to the corresponding low-dose inputs (**b**,**d**), respectively. The red arrows highlight specific regions where noise reduction in metal artefacts can be appreciated in the processed images. Although both cases (**a**,**c**) show some degree of noise reduction, as indicated by the arrows, the extent and quality of the improvement vary between the two scenarios.

To evaluate ErisNet’s behaviour in complex clinical scenarios, Figure 7 illustrates the model’s performance on CT images containing metal artefacts. It should be noted that the training dataset contained a limited number of cases with metal implants, which may be insufficient to allow the model to learn optimal denoising strategies in the presence of such artefacts. The results reported here suggest inconsistent performance, with the network appearing to respond differently depending on the extent of the metal artefacts present. These results indicate that the reliability of the model in cases with significant metal artefacts remains uncertain and would require dedicated training with a more representative dataset of metal artefact cases to achieve consistent clinical performance.

**Figure 8:**
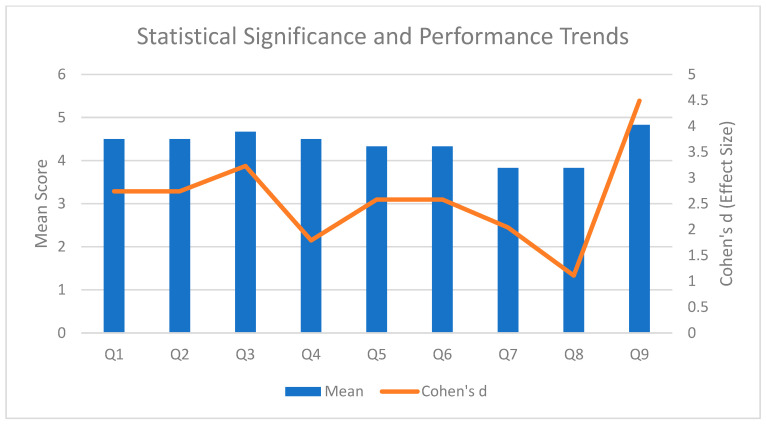
Statistical Significance and Performance Trends. Combined graph superimposing average scores and effect size. The blue bars show the average scores for each question, while the red line represents Cohen’s d values.

**Figure 9:**
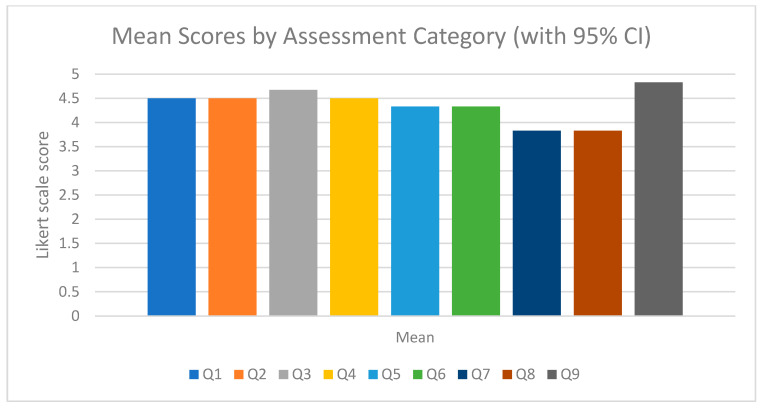
Average Scores by Assessment Category (with 95% CI). Histogram showing the average scores obtained for each question in the qualitative questionnaire, based on a Likert scale from 1 to 5. The error bars represent the 95% confidence interval (CI), indicating the precision of the mean estimate. The red dotted horizontal line at y = 3.0 represents the neutrality threshold.

**Figure 10:**
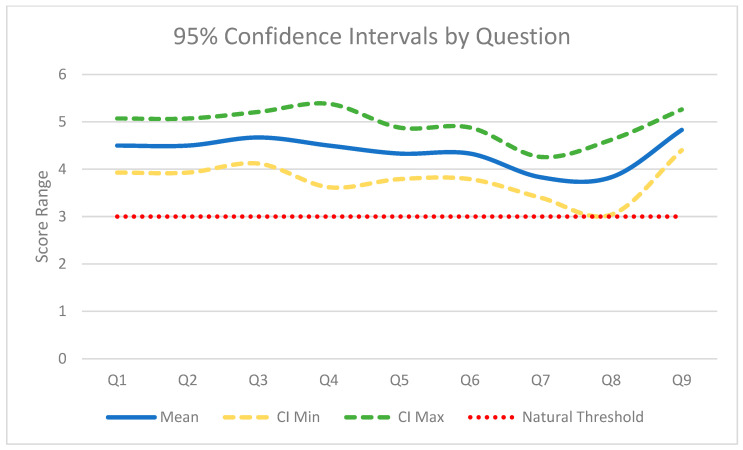
95% Confidence Intervals for Questions. Line graph showing the average score, upper confidence interval (CI Max), and lower confidence interval (CI Min) for each question. The green and yellow dotted lines represent the upper and lower limits of the confidence interval, respectively, while the red dotted line at y = 3.0 indicates the neutral threshold.

The graphs (Figure 8, Figure 9 and Figure 10) provide a detailed visual overview of performance trends and statistical significance.

## 4. Discussion

This study demonstrates that ErisNet significantly reduces noise in low-dose CT images while preserving diagnostic details. In the global quantitative analysis, the network achieved MSE = 64.07 ± 46.81, PSNR = 31.32 ± 3.69 dB, SSIM = 0.93 ± 0.06, VIF = 0.49 ± 0.09, EPI = 0.97 ± 0.01, and NV = 64.69 ± 46.80, a clear improvement over LQ reconstructions. ROI analysis confirmed noise suppression at the organ level: the SD of HU decreased by 7.95% in the vitreous (from 17.60 to 16.20 HU), by 18% in the brain parenchyma (from 18.85 to 15.40 HU) and to a similar extent in the liver, the spleen and muscles, with mean value changes < 2 HU in all regions. The qualitative assessment carried out by six radiologists produced scores ranging from 3.83 to 4.83 out of 5, with statistically significant improvements compared to LQ images (Holm *p* ≤ 0.042) and large effect sizes, particularly for the reduction in perceived noise (mean = 4.67, d = 3.23) and overall diagnostic confidence (mean = 4.83, d = 4.49).

When these results are placed in the context of the recent literature, it can be seen that ErisNet offers performance equal to and, in several parameters, superior to that of deep learning-based denoisers evaluated by Eulig et al. [13], a study that reported only marginal progress in the last six years for methods such as RED-CNN, Q-AE, BM3D, Noise2Void, and GAN-based approaches. From an architectural point of view, ErisNet combines a basic encoder–decoder structure with DnCNN-style residual blocks, exploiting multiscale feature aggregation via skip connections and aggressively attenuating high-frequency noise, thus resolving the typical trade-off between denoising strength and detail preservation reported for single-scale CNNs. Its training on paired high/low-dose PMCT images, made possible by the absence of radiation protection constraints, provides realistic noise statistics that are lacking in most studies based on phantoms or synthetic noise, potentially explaining the strong generalization observed in the present work.

## 5. Conclusions

This study presents several significant strengths that reinforce the validity and impact of the obtained results. First, the use of real PMCT data represents a substantial methodological advantage compared to most studies based on synthetic data or phantoms. This choice allowed us to obtain paired high- and low-dose images from the same subject, providing realistic noise statistics that are often absent in approaches based on artificial noise. This methodological strategy may explain the strong generalization capability observed in this work. Second, ErisNet’s hybrid architecture, which combines an encoder–decoder structure inspired by U-Net with DnCNN-style residual blocks, represents an innovative approach that effectively resolves the typical trade-off between denoising intensity and preservation of diagnostic details. Multi-scale feature aggregation through skip connections and aggressive attenuation of high-frequency noise distinguishes this approach from traditional single-scale CNNs. Third, the multidimensional evaluation conducted through three complementary approaches (global quantitative analysis using six image quality metrics, organ-level ROI analysis in five anatomical locations, and qualitative evaluation by six expert radiologists) provides robust and clinically relevant validation of the results. The convergence of results across these different evaluation methods significantly strengthens the credibility of the findings. Fourth, systematic hyperparameter optimization through Bayesian search algorithms (Hyperopt) followed by targeted fine-tuning ensured optimal model configuration, minimizing the risk of overfitting and maximizing system robustness. Fifth, the diversified dataset comprising 23 whole-body scans with a wide range of body dimensions (heights from 142 to 204 cm, BMI 18–32) and an average dose reduction of 55% (range 41–83%) demonstrates the model’s applicability across different anatomies and acquisition protocols.

Despite these strengths, several limitations remain and suggest directions for future research. First, ErisNet was only compared to iterative reconstruction and not to other contemporary deep learning-based reconstruction pipelines known to further reduce noise and artefacts [25,26]. Future studies should include comprehensive comparisons with state-of-the-art deep learning methods. Second, the evaluation set contained few metal implants, so robustness to metal-induced artefacts was not adequately tested. Future work should evaluate performance in the presence of various metallic implants commonly encountered in clinical practice. Third, performance was not stratified according to body habitus, although patient size influences noise behaviour [27]. Future investigations should assess ErisNet’s performance across different patient sizes and body compositions. Fourth, only one soft tissue reconstruction kernel was considered; kernel-specific tuning may improve results. Future developments should explore optimization for different reconstruction kernels and imaging protocols. Fifth, seamless integration into clinical workflows still requires validation of PACS compatibility and inference speed [25]. Future work should focus on clinical implementation aspects including real-time processing capabilities (ErisNet takes an average of 12 min to process a complete CT scan consisting of approximately 3300 images) and integration with existing radiological infrastructure.

The field of CT image denoising is rapidly evolving with emerging technologies that offer promising avenues for further research. Diffusion models represent a particularly exciting direction, as recent studies have demonstrated their potential for medical image reconstruction and denoising [28,29]. These probabilistic models excel at generating high-quality images while preserving anatomical structures, making them well-suited for low-dose CT applications where maintaining diagnostic quality is paramount [30]. Transformer architectures also show significant promise for CT denoising applications. The self-attention mechanism inherent in transformers enables effective capture of long-range dependencies in medical images, which can be particularly beneficial for noise reduction while preserving fine anatomical details [31]. Recent work has demonstrated that transformers can achieve superior performance in various medical imaging tasks compared to traditional convolutional approaches [32]. Hybrid approaches combining CNNs and Transformers represent another promising research direction. These architectures leverage the local feature extraction capabilities of CNNs with the global context modelling strengths of transformers, potentially offering optimal solutions for CT denoising [33,34]. Such hybrid models could address the limitations of purely convolutional or purely attention-based approaches by combining their respective advantages. Future iterations of ErisNet could benefit from incorporating these emerging technologies, potentially through ensemble approaches or by developing novel architectures that integrate diffusion processes, transformer attention mechanisms, and convolutional operations within a unified framework.

Overall, quantitative metrics, ROI results, and expert evaluations indicate that ErisNet can enable substantial dose reduction while maintaining or even increasing diagnostic reliability, representing a significant step towards the routine clinical adoption of neural networks for Low-Dose CT reconstruction.

## Figures and Tables

**Table 1:** Summary of previous CT image denoising studies and their limitations.

Study	Technique/Approach	Key Features	Limitations
Sadia et al. [7]	Denoising convolutional neural networks	Convolutional approach for image denoising	Requires trade-off between noise suppression and preservation of diagnostic details
Chen et al. [8]	Iterative denoising technique	Exploits spatial redundancy in the direction of increasing slice thickness	Tested only on phantom experiments
Liu et al. [9]	Physical model integrated with unsupervised learning	Uses generative neural networks trained on CT scans of phantom at different doses	Based on phantom data, may not represent real clinical scan complexities
Won et al. [10]	Self-supervised learning method	Generates pseudo-CT image pairs by adding artificial noise	Uses artificial noise, may not reflect real noise characteristics
Zhang et al. [11]	Unsupervised method	Exploits similarity between adjacent slices in thin-layer CT scans, trained with artificial noise data	Based on artificial noise data, limited to thin-layer CT applications
Zainulina et al. [12]	Self-supervised physics-based denoising	Does not require high-dose CT images as ground truth, employs simulated noise for training	Uses simulated noise, may not capture real-world noise variations
Eulig et al. [13]	Comprehensive review of CT denoising methods	Systematic analysis of various denoising approaches including deep learning methods for image quality improvement and dose reduction	Review paper—does not propose new methodology, focuses on existing techniques
U-Net [14]	Encoder–decoder architecture	Established deep learning architecture for image segmentation and denoising	Requires trade-off between noise suppression and preservation of diagnostic details
DnCNN [15]	Denoising convolutional neural networks	Convolutional approach for image denoising	Requires trade-off between noise suppression and preservation of diagnostic details

**Table 2:** CT acquisition parameters.

	High Dose				Low Dose			
n.	kV	mAs	CTDIvol (mGy)	DLP (mGy·cm)	kV	mAs	CTDIvol (mGy)	DLP (mGy·cm)
1	120	285	19.30	3349.9	100	80	3.16	548.10
2	120	371	25.09	4459.7	80	50	0.93	165.40
3	120	345	23.35	4191.5	80	80	1.49	270.60
4	120	328	22.20	3915.9	100	321	12.69	2238.70
5	120	394	26.68	5011.00	120	200	13.53	2542.00
6	120	364	24.65	4874.00	120	142	9.67	1911.00
7	120	432	29.29	5685.00	100	280	11.53	2230.00
8	140	246	29.80	5950.00	120	137	11.60	2315.00
9	120	234	19.70	3295.00	100	355	18.50	3019.00
10	140	231	28.00	5079.00	100	307	16.00	2848.00
11	140	255	30.90	5796.00	100	341	17.90	3366.00
12	120	247	20.80	3574.00	120	63	5.30	905.00
13	140	250	30.30	5482.00	140	125	15.20	2727.00
14	140	328	39.90	6314.00	140	137	17.00	2653.00
16	120	209	26.10	4989.00	120	118	10.10	1896.00
17	120	205	17.20	3233.00	120	75	6.31	1207.00
18	140	309	22.80	3993.30	140	97	11.80	2226.00
19	120	294	24.70	4357.00	120	113	9.49	1647.00
20	140	295	35.80	7033.00	140	119	14.50	2840.00
21	140	220	26.80	5176.00	120	81	6.94	1338.00
22	120	221	14.9	2784.00	110	56	3.79	704.00
23	140	223	27.3	5133.00	140	57	7.02	1321.00

Table 2 provides a detailed overview of the two distinct computed tomography (CT) scanning protocols used in this study, classified as high-dose and low-dose. The table compares the technical parameters and resulting radiation dose metrics for each protocol in patient scans. For each scan (n), the table lists the key acquisition parameters: tube voltage (kV) and tube current–time product (mAs). These parameters were set to provide either high or low radiation emission. The impact of these settings is quantified using two standard dose indices: the volumetric computed tomography dose index (CTDIvol) in milligray (mGy) and the dose-length product (DLP) in milligray-centimetres (mGy⋅cm). The data show that the low-dose protocol consistently resulted in significantly lower radiation exposure. Specifically, the CTDIvol and DLP values for the low-dose protocol were substantially reduced compared to the high-dose protocol.

**Table 5:** Qualitative analysis results.

	Q1	Q2	Q3	Q4	Q5	Q6	Q7	Q8	Q9
Mean	4.5	4.5	4.67	4.5	4.33	4.33	3.83	3.83	4.83
Median	4.5	4.5	5	5	4	4	4	4	5
SD	0.55	0.55	0.52	0.84	0.52	0.52	0.41	0.75	0.41
IQR	1	1	0.75	0.75	0.75	0.75	0	0.75	0
Standard error	0.22	0.22	0.21	0.34	0.21	0.21	0.17	0.31	0.17
Critical t value	2.57	2.57	2.57	2.57	2.57	2.57	2.57	2.57	2.57
CI width	0.57	0.57	0.54	0.88	0.54	0.54	0.43	0.79	0.43
CI minimum	3.93	3.93	4.12	3.62	3.79	3.79	3.40	3.04	4.40
CI maximum	5.07	5.07	5.21	5.38	4.88	4.88	4.26	4.62	5.26
t statistic	6.71	6.71	7.91	4.39	6.32	6.32	5.00	2.71	11.00
Raw *p* value	0.001	0.001	0.001	0.007	0.002	0.002	0.004	0.042	0.0001
Holm *p* value	0.007	0.007	0.004	0.014	0.007	0.007	0.012	0.042	0.001
Cohen’s d	2.74	2.74	3.23	1.79	2.58	2.58	2.04	1.11	4.49

Table 5: Summary of the qualitative assessment by six radiologists (Likert 1–5) of ErisNet images: for each question Q1–Q9, the mean, median, variability, 95% confidence interval, statistical significance (t, *p*, *p* Holm) and effect size (Cohen’s d) are reported.

## Data Availability

The datasets used and analyzed during the current study are available from the corresponding author on reasonable request and according to specific national regulations on data sharing.

## Abbreviations

The following abbreviations are used in this manuscript:

BMI	Body mass indices
CI	Confidence interval
CT	Computed tomography
CTDIvol	CT dose index
DLP	Dose length product
DnCNN	Denoising convolutional neural network
EN	ErisNet
EPI	Edge preservation index
HQ	High quality
IQR	Interquartile range
LQ	Low quality
MSE	Mean squared error
NV	Noise variance
PMCT	Post-mortem CT
PSNR	Peak signal-to-noise ratio
ReLU	Rectified Linear Unit
ROI	Regions of Inerest
SD	Standard deviation
SSIM	Structural similarity index measure
VIF	Visual information fidelity
